# Automatic Vertebral Column Extraction by Whole-Body Bone SPECT Scan

**DOI:** 10.1155/2013/647548

**Published:** 2013-04-10

**Authors:** Sheng-Fang Huang, Hao-Yu Chao, Pan-Fu Kao, Wei-Chih Shen, Yu-Hsiang Chou, Shu-Hsin Liu

**Affiliations:** ^1^Department of Medical Informatics, Tzu Chi University, Hualien 97004, Taiwan; ^2^Institute of Medical Sciences, Tzu Chi University, Hualien 97004, Taiwan; ^3^School of Medicine, Chung Shan Medical University, Taichung 40201, Taiwan; ^4^Department of Nuclear Medicine, Chung Shan Medical University Hospital, Taichung 40201, Taiwan; ^5^Department of Computer Science and Information Engineering, Asia University, Taichung 41354, Taiwan; ^6^Department of Nuclear Medicine, Buddhist Tzu Chi General Hospital, Taipei Branch, New Taipei City 23142, Taiwan; ^7^Department of Nuclear Medical, Buddhist Tzu Chi Hospital, Hualien 97004, Taiwan; ^8^Department of Radiological Technology, Tzu Chi College of Technology, Hualien 97005, Taiwan

## Abstract

Bone extraction and division can enhance the accuracy of diagnoses based on whole-body bone SPECT data. This study developed a method for using conventional SPECT for automatic recognition of the vertebral column. A novel feature of the proposed approach is a novel “bone graph" image description method that represents the connectivity between these image regions to facilitate manipulation of morphological relationships in the skeleton before surgery. By tracking the paths shown on the bone graph, skeletal structures can be identified by performing morphological operations. The performance of the method was evaluated quantitatively and qualitatively by two experienced nuclear medicine physicians. Datasets for whole-body bone SPECT scans in 46 lung cancer patients with bone metastasis were obtained with Tc-99m MDP. The algorithm successfully segmented vertebrae in the thoracolumbar spine. The quantitative assessment shows that the segmentation method achieved an average TP, FP, and FN rates of 95.1%, 9.1%, and 4.9%. The qualitative evaluation shows an average acceptance rate of 83%, where the data for the acceptable and unacceptable groups had a Cronbach's alpha value of 0.718, which indicated reasonable internal consistency and reliability.

## 1. Introduction

In nuclear medicine, bone scintigraphy is widely used to detect bone metastases [1]. Technetium-99m methylene diphosphonate (Tc-99m MDP) scanning is the most common method of staging bone metastases and osteomyelitis in routine examinations. In a common whole-body bone scan, planar image scan protocol is usually used because it is time-saving. However, overlapping objects in a planar image may cause difficulty in interpreting images. Single photon emission computed tomography (SPECT), a functional imaging method of reconstructing skeletal objects in three-dimensional (3D) from different projection views, provides more diagnostic information compared to planar imaging [2, 3]. Vertebral SPECT is also superior for detecting metastatic foci [4].

When using SPECT for bone scan, segmentation is essential for volume rendering and for perceiving overall spatial relationships before extracting bones of interest for further analysis. After segmentation, skeletal structures are usually labeled so that abnormalities can be located automatically. However, the significant noise and relatively poor spatial resolution of SPECT often degrade nuclear imaging quality, which makes labeling difficult [5]. In whole-body bone scan images, the Tc-99m MDP uptake can also be seen in some organs. The image contains not only bone sections but also some parts of soft tissues. To ensure adequate specificity, an automatic lesion detection algorithm is needed to distinguish soft tissues from skeletal structures. Additionally, image brightness could be irregular for different bone sections. Therefore, optimizing the global threshold for image intensity is difficult when extracting skeletal structures. Moreover, SPECT imaging provides a unique challenge in this issue because the 3D process for a SPECT image is time-consuming. Thus, an automated computer-aided detection/diagnosis (CAD) system is urgently needed to increase efficiency and effectiveness in bone metastasis screening.

So far there have been several studies proposed to solve segmentation problem in whole-body bone scan images. One of the simplest proposed solutions for solving the segmentation problem in whole-body bone scanning is binary classification based on gray level, such as thresholding or region growing. Erdi et al. proposed a semiautomated approach for quantifying the area of bone metastases [6]. After the user selects a seed point for each lesion, the procedure automatically extracts the abnormality and calculates the percentage of lesion involvement in each bone. The main contribution of this approach is saving physician's time on drawing region of interest (ROI). Yin and Chiu considered taking experts' knowledge into account to deal with segmentation problem [7]. Their approach to bone scintigraphy used a knowledge-based bone division method to detect abnormalities by performing fuzzy analysis of asymmetries in bone morphology. Although this approach eventually achieved good performance in hand and leg regions, it performed poorly when analyzing bones in the head and vertebral column. In Šajn et al., the extreme edges of the main skeletal regions were used as reference points [8]. Their study demonstrated that the obtained reference points are helpful for skeletal segmentation in bone scintigraphy. Sadik et al. proposed a procedure for building a decision-support system [9]. Abnormalities extracted according to threshold values were classified on a scale from 0.0 to 1.0 by an artificial neural network analysis of 14 clinical features. However, this approach could not detect areas with high symmetry and high uptake such as joints and vertebrae. Huang et al. proposed a CAD system for using a hybrid method of bone division to analyze planar whole-body bone images [10]. Fuzzy set histogram thresholding technique was used to differentiate bones from soft tissues. Morphological knowledge was then applied to preform skeleton segmentation. Finally, a bandwidth concept was introduced to detect hot spots. The overall sensitivity was 92.1% with 7.58 false detections per image.

**Figure 1:**
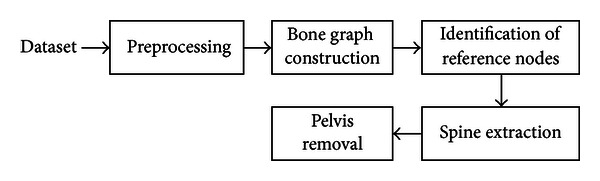
The flowchart of the proposed method.

In recent decades, the development of CAD systems for planar nuclear medicine imaging has steadily improved in terms of automation and sensitivity. However, fully automatic segmentation of whole-body bone SPECT scan images is still an open issue, and relevant CAD applications are quite limited in number. He et al. reported an image intelligent system that provides automatic diagnosis to process whole-body bone SPECT images [11]. The method employs optimal thresholding algorithm to extract bone regions from background and uses histogram equalization to reduce the effect caused by the high uptake of bladder. However, since the system does not contain any function for bone division to provide skeletal information, the system cannot automatically determine the positions of abnormalities and may fail to reduce the ambiguity between bone lesions and normal parts, such as joints and kidneys. Under this consideration, our study extended a previous version of our CAD system developed in 2009 [12] and designed a 3D segmentation method for using whole-body bone SPECT images for structural analysis of human skeletal anatomy. In this paper, we focus on the techniques of spine extraction, owing to the reason that the vertebral column affects up to a third of all cancer patients and is the most frequent site of metastatic involvement of the skeleton [13]. This study developed a novel “bone graph” data structure for formulating bone segmentation problems as a graph clustering problem. The study includes quantitative and qualitative assessments to evaluate the performance.  Figure 1 shows a flowchart of the proposed method.

## 2. Data Acquisition

The datasets were collected for 46 lung cancer patients with bone metastasis who had received Tc-99m MDP whole-body bone scans by SPECT at Buddhist Tzu Chi Hospital, Taipei Branch. The SPECT images for all patients were reviewed by a single experienced nuclear medicine physician. Patients with renal abnormalities and patients who had received surgical implants containing cement, rods, or screws were excluded from the study. A GE Infinia Hawkeye 4 gamma camera was used to acquire data in 6 degrees and with 16 seconds per step. One detector head collected data in 180 degree and another one collected data in the opposite 180 degree [14]. The SPECT imaging started at 3.5 hours after tracer injection. A full scan requires 5 sections, taking 8 minutes for each. The scan time was 40 minutes in total. The projection data were reconstructed using the GE's Evolution for bone reconstruction algorithm, where a 3D Collimator-Detector Response compensation method is used and integrated into an iterative reconstruction algorithm for SPECT images. Each dataset was saved in Interfile (Analyze 7.5) format. Image slices were included in a single image file and other information were saved in a header file. This study was reviewed and approved by the institutional review board at Tzu Chi Hospital. This Health Insurance Portability and Accountability Act-compliant single center is a retrospective study. A waiver of informed consent was obtained because the research involves no more than minimal risk and the waiver will not adversely affect the rights and welfare of the patients.

## 3. Method

### 3.1. Preprocessing

**Figure 2:**
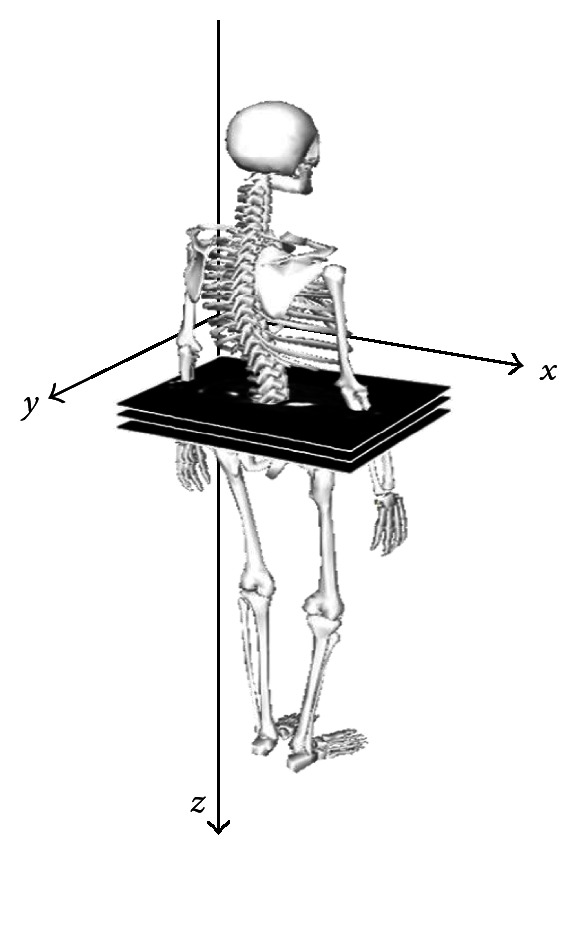
The three axes of the coordinate system defined in the proposed method.

A dataset containing whole-body bone scan SPECT images is initially converted to bitmap format images in transaxial view. Assume that the coordinate system of the images is defined as shown in Figure 2. A threshold value *T* is then applied to separate bone regions from soft tissues. However, the partial-volume effect (PVE) could lead to lower intensity values for voxels that only partially represent bone regions. Therefore in this study, moment-preserving thresholding is used to determine the value of *T* [15]. The thresholding method was computed based on the image histogram improved by using boundary characteristics, in order to avoid the interference caused by a large background area or the extremely high intensities from disease hotspots [16]. The image is then converted into a binary image, *b*(*x*, *y*, *z*), where *b*(*x*, *y*, *z*) equals 1 if the pixel value is larger than; otherwise, 0 is assigned. For each image slice, a connected component with gray level higher than *T* is defined as bone region. Each region is characterized by its shape features, including spatial center, area and bounding box, which will be used to determine the topological relationships among regions.

### 3.2. Design of Bone Graph Model

#### 3.2.1. Graph Initialization

A special graph is used to describe the connectivity between bone regions so that segmentation problems could be solved with graph algorithms. This “bone graph” can be represented by *G* = (*V*, *E*), where *V* denotes the node set and *E* denotes the edge set. The elements of *V* and *E* have the following characteristics.

1. Each region (connected component) in each plane is represented by a node in *V*.
2. When a plane *K* contains a node *m*, a plane *L* contains a node *n*, and *K* < *L*, a directed edge extending from *m* to *n* (down-link) and a directed edge extending from *n* to *m* (up-link) are added to *E* if a projection of node *m* onto plane *L* overlaps with node *n*.

**Figure 3:**
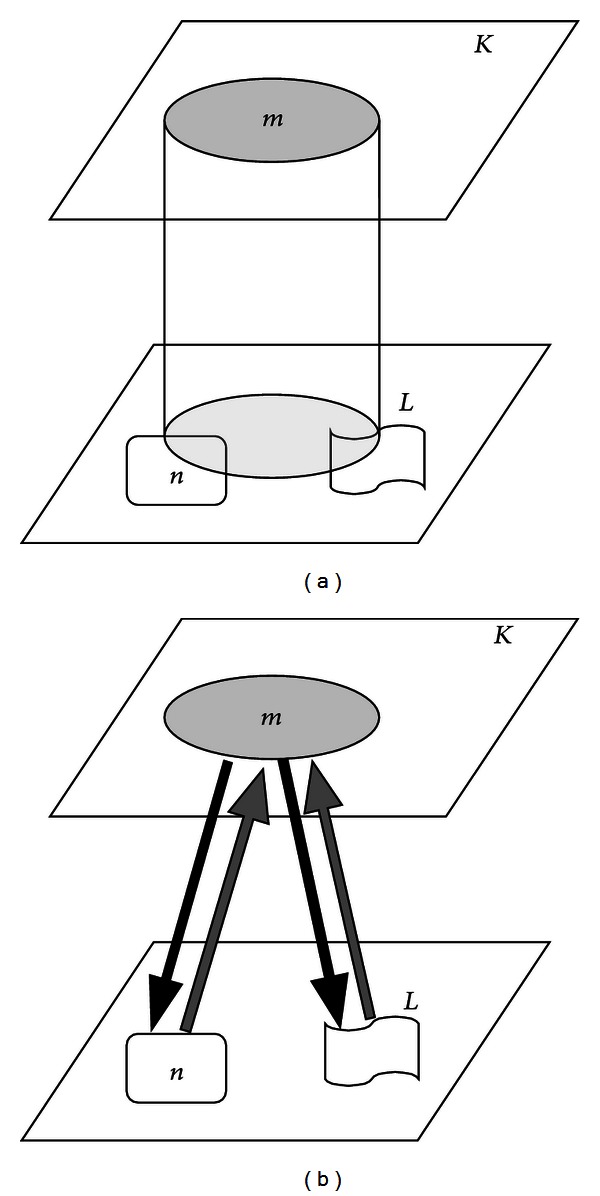
The construction of a bone graph. If the region *m* in the plane *K* overlaps the region *n* in the plane *L*, and then a downlink edge pointing from *m* to *n* and an uplink edge from *n* to *m* (uplink) are added.

The construction of the bone graph is shown in Figure 3, where the node level is determined by the number of slices to which it belongs. The discrimination of uplink and downlink can provide a clear definition to the edge direction on the graph, which indicates a super-/subordinate relation link between two nodes. For example, a node *p* pointed by an uplink from a node *q* implies that the plane where *p* is located is higher than that of *q*. This prevents the traverse from falling into the wrong level of image plane when nodes are scanned in the upward or downward direction. Each edge is weighted in order to evaluate the correlation between neighboring nodes. If *R*(*i*) is the set of projected pixels corresponding to the node *i*, the edge weight, *w*_*ij*_, represents the region similarity of the node *i* with the node *j* and can be defined as follows:

(1) $$\begin{array}{c} w_{ij}=\frac{\left|R\left(i\right)\right|\left|R\left(i\right)\cap R\left(j\right)\right|}{{\left|R\left(i\right)\cup R\left(j\right)\right|}^{2}}. \end{array}$$

**Figure 4:**
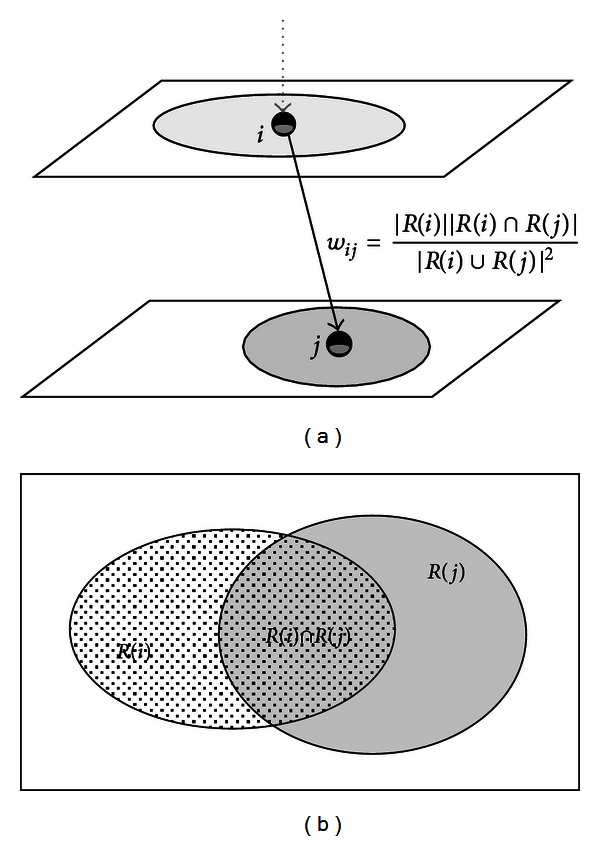
The edge weight *w*_*ij*_ is computed as the similarity evaluation between two image regions. The value is based on the fraction of how large the two regions overlap on the 2D projection plane, as shown in (b).

Varying from 0 to 1, a significant overlap of *R*(*i*) with *R*(*j*) will result in a high value of *w*_*ij*_, as shown as Figure 4.

#### 3.2.2. Model Optimization

Given the pairwise similarity matrix **W** = (*w*_*ij*_), segmentation using the bone graph can be modeled by a graph-cut clustering framework. The similarity between two clusters *C*_1_ and *C*_2_ is defined as follows:

(2) $$\begin{array}{c} S\left(C_{1},C_{2}\right)\equiv \sum_{i\;\in \;C_{1}}\sum_{j\;\in \;C_{2}}w_{ij}. \end{array}$$

The objective of the proposed segmentation method requires an iterative process of image region decomposition and graph modification so that *S*(*C*_1_, *C*_2_) is minimized to zero. The algorithm is described next.

**Step 1**

At the beginning, a seed node is selected into a cluster denoted by *C*_1_, while each of the other nodes is a cluster itself, denoted by *C*_*i*_ for *i* > 1:

**Step 2**

For all *i* > 1, if *S*_1*i*_ = *S*(*C*_1_, *C*_*i*_) > 0, the distance from *C*_1_ to *C*_*i*_ is measured by the following cost function:

(3) $$\begin{array}{c} d_{i}=\frac{\left|S_{1i}-\sum_{j>1,j\neq i}S_{ij}\right|}{\max\left(S_{1i},\sum_{j>1,j\neq i}S_{ij}\right)}. \end{array}$$

If *d*_*i*_ < 0.5, then *C*_*i*_ is combined into *C*_1_; otherwise, *C*_*i*_ is split to create more clusters. To achieve this, dame pixels are built by using morphological operations to decompose the corresponding image region into parts. The bone graph is then updated to reflect this modification.

**Step 3**

If ∑_*i*>1_ *S*_1*i*_ = 0, the process stops. Otherwise, it goes back to Step 2 to continue.

**Figure 5:**
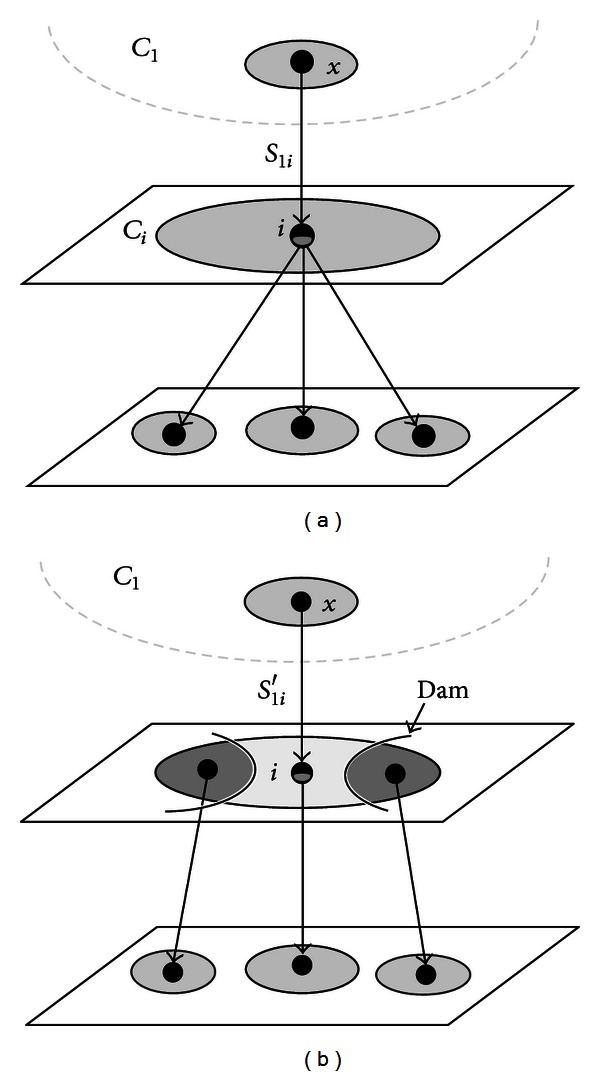
(a) The pattern represents an unbalanced cluster *C*_*i*_, which usually corresponds to a branching node in the graph. (b) A cluster splitting procedure creates dame lines to separate the image region of the node *i* into more parts. After that, the bone graph and its corresponding clusters are updated so that the merging process can be continued.

Figure 5 illustrates a situation that requires cluster splitting. The graph pattern shown in this figure may occur at a branching node, such as shoulders, joints, or a vertebrae nearby kidneys, which will result in irregular similarities with neighboring clusters. The distance computed by the cost function is used to evaluate this variance in order to determine whether the expansion of *C*_1_ would be trapped into an unbalanced cluster. If so, a cluster splitting process is then performed to modify the graph topology. At the end when ∑_*i*>1_ *S*_1*i*_ = 0, the subgraph included in the cluster of *C*_1_ is entirely isolated and disconnected to other subgraphs, where the image regions of the extracted subgraph form the final segmentation result of interest.

#### 3.2.3. Image Decomposition and Cluster Splitting

To decompose a target region image *M*, a set containing *n* reference regions is defined as *R*_*M*_, where *R*_*M*_ = {*M*_*k*_ | *M*_*k*_⊆*M*  and  *M*_*x*_∩*M*_*y*_ = *ϕ*  for  1 ≤ *x*, *y* ≤ *n*  and  *x* ≠ *y*}. To split *M*, each *M*_*k*_ is iteratively enlarged by using morphological dilation. The dilation is restricted within the bound of *M* and continues until the entire area of *M* is filled. At each iteration, the coordinates where two dilated regions met are marked as dam points. Once this procedure completes, the dam points are removed, which therefore separates *M* into *n* subregions.

According to the algorithm in Section 3.2.2, if a clustering splitting procedure is required for *C*_*i*_, we decompose the image region of *C*_*i*_ in order to create more clusters. Suppose that the image region of *C*_*i*_ is defined as *M*. As illustrated in Figure 5(b), if the node *x* in *C*_1_ connects to the node *i* of *C*_*i*_, then assume that *R*(*x*) denotes the set of projected pixels corresponding to the node *x*. The set of reference regions, *R*_*M*_. can be assembled by the following regions:

(4) $$\begin{array}{c} R_{M}=\left\{\left[R\left(x\right)⊕B\right]\cap M\right\}\cup \left\{r\;|\;r\subseteq \left[M-R\left(x\right)\right]⊗B\right\}. \end{array}$$

The symbols ⊗ and ⊕ represent image morphological operators, respectively, for erosion and dilation. The term *B* is a 5 × 5 window, denoting the structuring element for the morphological operations. The image region of *M* is then decomposed based on *R*_*M*_. Finally, the nodes and edges of the bone graph, as well as node clusters, are therefore updated according to the new topology.

### 3.3. Details of the Proposed Method

#### 3.3.1. Identifying Reference Nodes

Two reference nodes are required as anatomical landmarks in the bone graph, including neck and hip-joint. To identify the reference node for neck, the top node of the graph is retrieved at first, and then the down-links are traced through each node in successive slices. For each traversed node, as the bounding box area of its corresponding region is examined, the maximum area is updated as a representative value of the cross-sectional area for head. Since the cross-sectional area of neck is relatively smaller than that of head, the reference node of neck, denoted as RP_Neck_, is marked if its corresponding region has the bounding box with area less than a threshold. In our study, the threshold value is determined based on human anatomy as well as the physicians' recommendations, which is 40% of the maximum cross-sectional area of head.

For the reference node of hip joint, the thresholded images computed in the preprocessing section are used to identify the nodes of two legs on the bone graph. First, pixels in the last third of the binary images were scanned. The counts of nonzero pixels were then computed over the *x*-axis as follows:

(5) $$\begin{array}{c} p\left(x\right)=\int_{n/3}^{n}\int_{y}^{}b\left(x,y,z\right)dy\;dz. \end{array}$$

**Figure 6:**
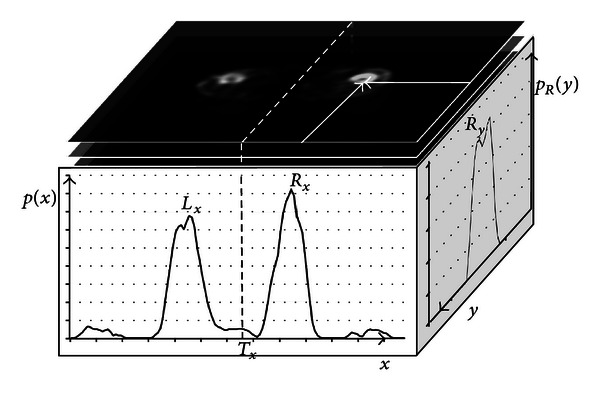
The accumulation of the horizontal counts of nonzero pixels from the bottom to the *k*th image plane where *k* is given.

As illustrated in Figure 6, the curve of *p*(*x*) has two peaks relatively corresponding to the positions of the two legs. Since the curve is highly symmetrical, the two groups can be separated by an optimal global threshold obtained by a binarization method [17]. In Figure 6, *L*_*x*_ and *R*_*x*_ indicate the coordinates with the maximum values in their respective groups on the *x*-axis. The ratios of nonzero pixel count over the *y*-axis, denoted as *p*_*L*_(*y*) and *p*_*R*_(*y*), are also recomputed. The *y* coordinates with the maximum values of *p*_*L*_(*y*) and *p*_*R*_(*y*) are, respectively, defined as *L*_*y*_ and *R*_*y*_. Next, by centering on the pixels at the coordinates of (*L*_*x*_, *L*_*y*_) and (*R*_*x*_, *R*_*y*_) on each image plane, two ROIs, *R*_LEFT_ and *R*_RIGHT_, respectively, are definable where the diameters of the ROIs were determined by the standard deviations computed from *p*_*L*_(*y*) and *p*_*R*_(*y*). The two ROIs are used to identify the leg nodes in the bone graph. For a node, if the corresponding region has the center locating within the ROI *R*_LEFT_ or *R*_RIGHT_, the node is then marked as left or right leg node. As tracing the uplinks for these nodes in the bone graph, the tracking procedure marks the traversed nodes on the bone graph as left or right leg nodes, which are then connected to form a tracking path for each leg. The procedure eventually identifies the node joining the two paths, which indicates the location of the hip joint. The node is then marked and designated the hip-joint reference node (RP_Hip-joint_).

#### 3.3.2. Spine Extraction

Given RP_Neck_ as the initial node in *C*_1_, additional spine nodes are found by traversing upward and downward in the graph to optimize the clustering of bone graph model, as described in Section 3.2.2. This process measures the cost for each node connecting to *C*_1_. Any node satisfying the cost criterion will be marked as a spine node and joined to *C*_1_. Also, as *C*_1_ expands, the image region decomposition and cluster splitting procedures are involved in order to disconnect the vertebral to other structures, such as rib-cage, joints, pelvis, or kidneys. The segmented image regions are then stacked into volumetric data. A morphological opening with a 3 × 3 × 3 structuring element is finally applied on the 3D image to smooth the segmentation result.

#### 3.3.3. Pelvis Removal

Residual urine activity in the bladder often produces interference in whole-body bone scan images. The bladder is close to the sacrum, and the urine activity usually has relatively higher pixel values compared to soft tissues. Therefore, the bladder may be misinterpreted as part of spine because of its location in a region connecting the vertebral column in the bone graph. Because the bladder is surrounded by the pelvis, the final step of spine segmentation is to exclude the section of pelvis to avoid containing the bladder in the final result. Two image planes are determined to evaluate the range of pelvis. First, the leftmost and rightmost coordinates on the *x*-axis for the regions corresponding to leg nodes are computed. Given the thresholded images as input, the pixels not within this range are excluded as background. In addition, the pixels of the regions corresponding to the nodes marked as spine or as legs are also eliminated from the images. In the image plane where the hip-joint reference node is located, the nonzero pixels are selected as initial seed points. A 3D region-growing segmentation algorithm is performed on the image stack to form a connected component for the pelvis [6]. On the *z*-axis, the upper and lower ends of the connected component are marked to generate a range for representing pelvis location. In the extracted segmentation result, any pixel labeled as spine within this range is removed.

### 3.4. Evaluating Segmentation Result

The study includes quantitative and qualitative assessments to evaluate the performance. For the quantitative assessment, segmentation accuracy was tested by comparing the automatically extracted vertebral column images to the ground truth. Since the datasets of the study do not have corresponding CT images as gold standard, the manual segmentation was obtained by image thresholding that followed a sequence of manual adjustments to remove the structures not belonging to vertebral column, where the optimal threshold value was chosen manually for each dataset. The accuracy was measured with three different error measures: true-positive (TP) and false-positive (FP) volume fractions, which were defined as follows [18]:

(6) $$\begin{array}{c} \text{TP}=\frac{\text{Volume}\left(A_{a}\cap A_{m}\right)}{\text{Volume}\left(A_{m}\right)},\\ \text{FP}=\frac{\left|\text{Volume}\left(A_{a}\cup A_{m}\right)-\text{Volume}\left(A_{m}\right)\right|}{\text{Volume}\left(A_{m}\right)},\\ \text{FN}=\frac{\left|\text{Volume}\left(A_{a}\cup A_{m}\right)-\text{Volume}\left(A_{a}\right)\right|}{\text{Volume}\left(A_{m}\right)}, \end{array}$$

where the term *A*_*m*_ refers to the vertebral regions determined by manual segmentation and *A*_*a*_ are the ones extracted by the proposed method.

For the qualitative evaluation, two experienced nuclear medical physicians independently examined the segmentation results. In this test, each dataset was classified “acceptable” or “unacceptable” by a physician after comparing the original images with those obtained by the computer. A dataset is classified as acceptable if only a few slices (fewer than 10%) had computed ROIs that did not perfectly match the boundary of spine bone region and in which the segmentation area would not substantially affect the future identification of lesions and other abnormalities. Finally, unacceptable segmentation denotes an error in ROI computed for skeletal structures or other organs (e.g., the kidney or bladder) that was large enough to produce a misdiagnosis. Finally, the acceptance rate of the method was quantified by the ratios of datasets that were classified “acceptable”.

**Figure 7:**
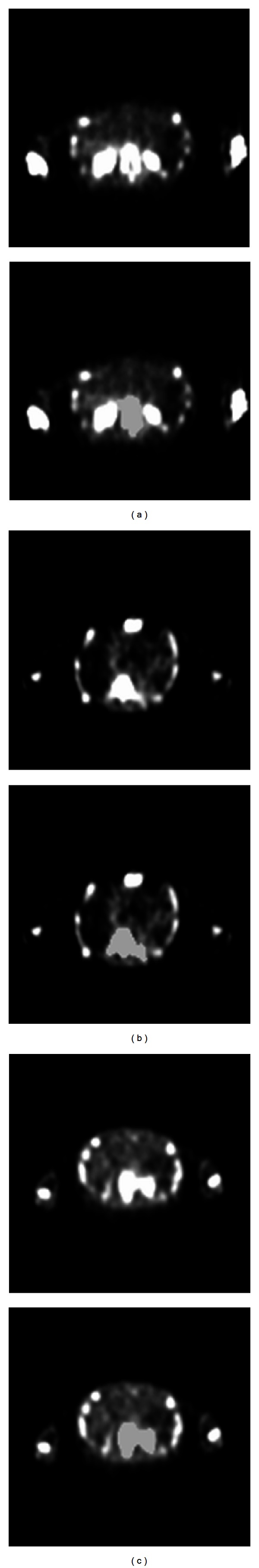
(a) and (b) show two examples of acceptable segmentation results and (c) illustrates an example of unacceptable result. The first row denotes the transaxial planes extracted from the original SPECT data, whereas the second row lists their corresponding segmentation results.

Figure 7 shows examples of segmentation results. The images in the first row denote the transaxial planes extracted from the original SPECT data, whereas the second row lists their corresponding segmentation results. The regions colored in light grey represent the segmented vertebrae regions.  Figure 7(a) shows a perfect segmentation result where bone regions can be successfully extracted and fully isolated from other skeletal structures and organs.  Figure 7(b) shows segmented bone regions partly covering soft tissues. These regions generally had lower intensities and were not considered hot spots. The dataset was also classified as acceptable segmentation since the partial obstruction and reduced intensity did not affect diagnostic accuracy. However, if the segmented area overlapped kidney regions as shown in Figure 7(c), the result was then classified as unacceptable.

To test the reliability of the independent evaluations by two different physicians, Cronbach's alpha values were computed for the two sets of grades. The Cronbach's alpha value is a statistical measure of the correlation between two groups of observed scores, which is used to determine the internal consistency between two observers [19]. That is, expert evaluations should be considered trustworthy only if their evaluations are adequately correlated. The equation for calculating Cronbach's alpha is

(7) $$\begin{array}{c} \alpha =\frac{k}{k-1}\left(1-\frac{\sum_{i}^{K}s_{i}^{2}}{s_{T}^{2}}\right), \end{array}$$

where *k* is the number of items, *s*_*i*_^2^ is the variance in the *i*th item and *s*_*T*_^2^ is the variance in the total score formed by summing all the items. In the study, *k* equals 46, which corresponds to the number of datasets. Each item was assigned a score of 0 or 1, to represent an unacceptable or acceptable segmentation result. The theoretical value of alpha value varies from zero to 1, where a higher value larger than 0.7 is desirable for an appropriate degree of reliability.

## 4. Results and Discussion

The area error metrics TP and FP can quantitatively evaluate the performance of the proposed method. The average TP, FP, and FN percentages obtained by the system were 95.1%, 9.1%, and 4.9%, respectively. For the qualitative assessment, Table 1 lists the ratios for the two categories assigned by the two physicians. According to the table, 89% and 76% (average, 83%) of all images were acceptable.

**Figure 8:**
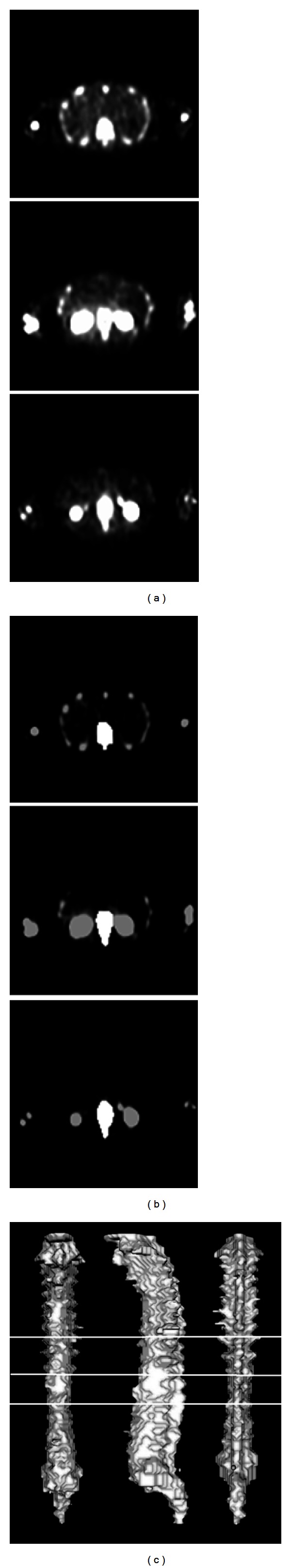
The reconstruction of the segmentation result classified as acceptable. (a) Original transaxial image plane, (b) segmented vertebral regions (white), and (c) the 3D reconstruction, where the white line denotes the height for the left three images in (a).

**Figure 9:**
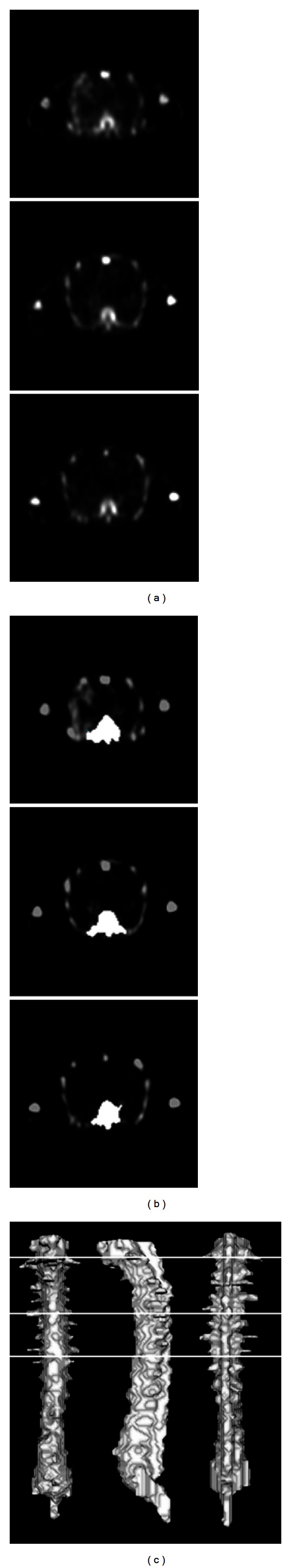
Another dataset that was also classified as acceptable. (a) Original transaxial image plane, (b) segmented vertebral regions (white), and (c) the 3D reconstruction, where the white line denotes the height for the left three images in (a).

**Figure 10:**
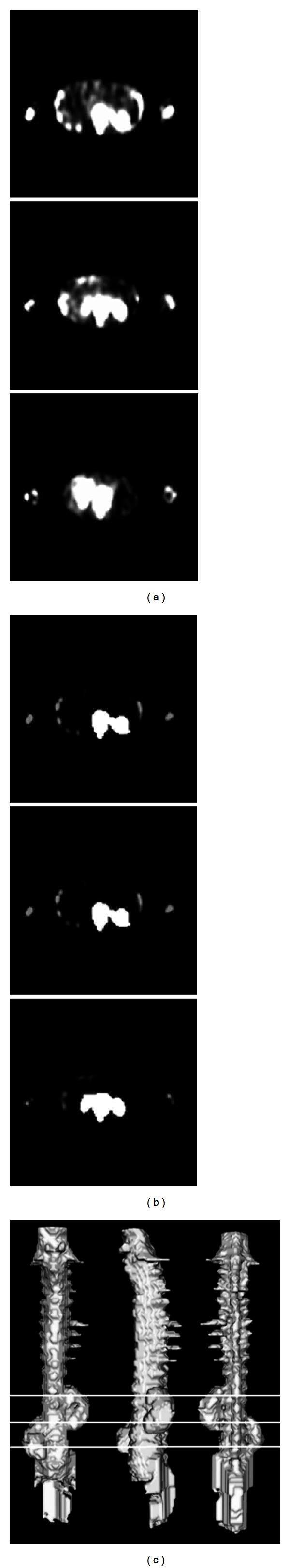
The reconstruction of the segmentation result classified as unacceptable. In this case, the kidneys are very close to the spine due to ectopia. (a) Original transaxial image plane, (b) segmented vertebral regions (white), and (c) the 3D reconstruction, where the white line denotes the height for the left three images in (a).

Figures 8–10 show three segmentation results, which were reconstructed by surface rendering using the Visualization ToolKit (VTK). Figures 8 and 9 demonstrate acceptable segmentation results, while Figure 10 shows an erroneous outcome for those classified as unacceptable. The first column in Figures 8–10 lists three transaxial slices extracted from their original SPECT data, and their positions are given in the third column. Figures 8 and 9 show successfully extracted vertebral columns in which high-activity organs such the kidney or bladder were fully excluded (images in second column). Figure 10 shows a case in which the horizontal boundary of one kidney is very close to the spine. The method initially grouped the bone and kidney as a single bone region, which produced errors in subsequent iterations of the segmentation procedure. The Cronbach's alpha values for the two sets of grades (0.718) exceeded 0.70, which indicated that the grades had reasonable internal consistency and reliability.

Factors that can degrade image quality and produce segmentation error [20–22] include equipment malfunctions such as those in a camera that has not been serviced regularly or the use of an inaccurate flood correction map, which can produce image noise and may cause a nonuniform appearance similar to that produced by soft-tissue uptake. Additionally, movement by the patient frequently causes blurred images, which may be misinterpreted as soft-tissue uptake. Finally, even when performed correctly, soft tissues may cause faulty uptake patterns when using the proposed method.

The degree of noise in SPECT images also varies due to the factors of different imaging protocols and reconstruction parameters and therefore could affect the accuracy of the method. So far the proposed method has only focused on how to delineate important landmarks on skeleton and extracting vertebral column from the original SPECT image. Therefore, the imaging protocol and scan time used in the study were managed to guarantee reduced noise in the image. In the proposed method, most noise in the background and soft tissues can be removed by the moment preserving method. In the future, a more complicated image filtering algorithm for noise reduction can be used in the preprocessing step if the other imaging protocols are considered. Comparison between different preprocessing algorithms and how they impact on the segmentation accuracy will also be analyzed.

## 5. Conclusions

Compared to planar bone scintigraphy, SPECT scan provides better contrast resolution without superposing body structures, which increases the specificity of positive scan findings and improves accuracy in locating equivocal lesions. Furthermore, an automated CAD system can effectively help doctors to identify whether a whole body bone SPECT scan is abnormal. As shown in the study of He et al. [11], bone lesions can be recognized according the morphological data of high uptake regions, but with the lack of spatial information, false-negatives may still remain due to the ambiguity of high uptake resulted by neighboring organs and skeletal joints. Combining functional data with anatomical findings should further increase its diagnostic accuracy. However, without the aid of an additional CT component, the anatomical information of bone scintigraphy acquired by SPECT is difficult to interpret due to the complexity of human skeletal structures. For conventional SPECT scan, this study developed a software solution for recognizing important anatomical landmarks on SPECT bone scintigraphy. To our knowledge, this study is the first to address this problem. The proposed “bone graph” image description method graphically depicts the connectivity between bone regions on neighboring images so that bone specialists can graphically manipulate the morphological relationships of skeletal structures. By using the graph to characterize the images, the problem of vertebral column extraction can be solved by applying graph algorithms.

Extracting the vertebral column is a challenging task because the spine is connected to many other skeletal structures such as the skull, shoulder bones, rib cage, and pelvis. By tracking their paths with the proposed bone graph, morphological operations can be performed to isolate these parts. For each dataset, the performance of the proposed method was confirmed by independent reviews of images by two experienced nuclear medicine physicians. According to the quantitative evaluation, the segmentation method achieved a high TP rate of 95.1% and a low FN rate of 4.9%, showing that most of the ROIs obtained automatically by the proposed method have a large percentage of overlapping to the manual segmentation. The FP rate was 9.1%, which was slightly higher than the FN percentage due to the segmentation that erroneously included kidneys and other skeletal structures. The qualitative assessment shows an average acceptance rate of 83%. The Cronbach's alpha value between the two sets of scores was 0.718, which indicated reasonable internal consistency reliability.

Although the algorithm successfully segmented vertebrae in the thoracolumbar spine, errors may occur in sections near the bladder. Because tracer not taken up by bones is excreted in the urine of a patient with normal renal function, the bladder and kidneys are overenhanced in a whole-body bone scan image, which may complicate the segmentation of vertebrae. High bladder activity reportedly renders up to 20% of SPECT pelvis scans unusable [23]. The proposed method, however, simply used the computed reference lines to define a VOI around the pelvis to exclude the bladder. Other techniques, such as atlas-based registration scheme [24], can be adopted to improve the segmentation accuracy. Nevertheless, this method may exclude lesions near the sacrum, which would increase the false-negative rate. The recent development of integrated hybrid SPECT/CT systems, which provide functional and anatomical images in the same scanning session, could significantly improve its diagnostic capability [25]. From the perspective of image engineering, both SPECT and CT require segmentation techniques for extracting the important anatomical information to achieve a precise image fusion. This study develops an approach in delineating human anatomy using SPECT images without the aid of CT. In the future, the anatomical information extracted by using the proposed image characterization technique can be combined with image registration and fusion method in the SPECT/CT system for improving anatomic localization of a suspected site of increased radiotracer uptake at bone scanning. The result of this study can be further applied on the detection and localization of bone metastases in vertebrae. In future studies of whole-body bone division, the authors will include hot spot detection in the proposed bone graph. Partial extraction of SPECT image data will enable investigators to exclude typical hot spots such as joints. In addition, contrast enhancement or thresholding approaches can be optimized for different skeletal sections to improve irregularly distributed intensity. For classifying bone metastases, the authors will also explore methods combining texture analysis with a machine-learning algorithm. The proposed segmentation method may also be used to increase diagnostic accuracy and efficiency in an automated CAD system for quantitatively estimating bone metastases.

## Figures and Tables

**Table 1:** Correct acceptance rates of the method.

	Physician 1	Physician 2	Average
Acceptable	41 (89%)	35 (76%)	83%
Unacceptable	5 (11%)	11 (24%)	17%

Total	46	46	
